# Cohabitation and marriage during the transition between adolescence and emerging adulthood: A systematic review of changes in weight-related outcomes, diet and physical activity

**DOI:** 10.1016/j.pmedr.2020.101261

**Published:** 2020-11-28

**Authors:** André O. Werneck, Eleanor M. Winpenny, Campbell Foubister, Justin M. Guagliano, Alex G. Monnickendam, Esther M.F. van Sluijs, Kirsten Corder

**Affiliations:** a MRC Epidemiology Unit and Centre for Diet and Activity Research (CEDAR), University of Cambridge, Cambridge, UK; b Department of Physical Education. Universidade Estadual Paulista “Júlio de Mesquita Filho” (UNESP), Presidente Prudente, Brazil

**Keywords:** Marriage, Adiposity, Exercise, Life transition

## Abstract

Our aim was to systematically review the effect of cohabitation and marriage on physical activity, diet and weight-related outcomes during emerging adulthood. A systematic search of six electronic databases was conducted until July 2019 (PROSPERO:CRD42018106943). Prospective studies were included if data were presented for a weight-related outcome, physical activity, and/or diet among 15–35 years-old participants assessed pre- and post-cohabitation or marriage and compared to a consistently non-cohabiting/non-married (single) reference group. Following title/abstract screening, two reviewers independently screened full-text and assessed risk of bias. There were 11 studies that met inclusion criteria. Outcomes included: body mass index (BMI) only (n = 3), physical activity only (n = 4), diet only (n = 2), BMI and physical activity (n = 1), and all outcomes (n = 1). Cohabitation or marriage was associated with greater BMI increases compared to remaining single among both men and women. Three studies analysed separately cohabitation and marriage and 3 of 4 found that only marriage was associated with higher BMI. Compared to being consistently single, starting cohabitation or getting married were associated with decreased physical activity in 2 of 4 studies among men and 4 of 6 studies among women, with no differences between marriage and cohabitation. Of the three studies examining change in diet, two showed no difference between individuals beginning to cohabit compared to those remaining single; without gender differences. Starting cohabitation and getting married may be valuable targets for weight management interventions, but more studies are needed to investigate the effect of cohabitation and marriage on health behaviours.

## Highlights

• Starting cohabitation and getting married were associated with increased BMI.
• Findings were mixed for the effect of starting cohabitation/marriage on PA.
• Limited evidence for the effect of starting cohabitation/marriage on diet.

## 1 Introduction

The prevalence of physical inactivity, poor diet quality and obesity is high worldwide (Guthold et al., 2018, Imamura et al., 2015, Ng et al., 2014), and previous evidence has demonstrated that the transition between adolescence and emerging adulthood is a period when the prevalence of obesity and obesity-related behaviours may increase particularly quickly (Corder et al., 2017, Harris et al., 2009, Winpenny et al., 2017). Previous cohort studies found that the period between 15 and 35 years is the one with higher increases in overweight and obesity rates (Johnson et al., 2015). The transition period from adolescence to emerging adulthood (i.e. 15–35 years) is also marked by several key life events including moving out of the family home, starting work, starting cohabitation, getting married and having children. These life events may lead to increased responsibilities and a change in priorities, consequently these events can have an impact on lifestyle behaviours and may provide opportunities for the improvement of health behaviours such as physical activity and diet (Corder et al., 2020, Rapp and Schneider, 2013, Winpenny et al., 2020).

Marriage has been shown to be protective for chronic diseases among middle-aged and older adults on studies linking data from census to later census surveys and death records (Blomgren et al., 2012, Grundy and Tomassini, 2010), which has been suggested to be due to a natural selection of peers, in which healthier people are more likely to have stable relationships with healthier people (Guner et al., 2018). Those in cohabiting relationships, particularly men, may also adopt less deviant behaviours, such as reducing excess alcohol consumption and tobacco smoking (Josefsson et al., 2018). However, marriage and cohabitation are correlated with increases in social responsibilities that can consistently reduce the amount of perceived leisure time available for conducting healthy behaviours, such as physical activity (Rapp and Schneider, 2013), and have previously been associated with increased risk for obesity among middle-aged adults (Cobb et al., 2016). The effect of marriage and cohabitation on behaviours may also differ across age groups. This is supported by previous research showing a negative effect of marriage and cohabitation on physical activity, which reduces and even becomes positive over the years, especially among men (Rapp and Schneider, 2013).

In contemporary Western societies, most young adults start cohabiting before entering formal marriage (Sassler and Lichter, 2020), and it is possible that the effect of cohabitation and marriage on behaviours and obesity can differ. For example, marriage can promote greater social and legal stability than cohabitation (Sassler and Lichter, 2020), which can be associated with positive health outcomes such as wellbeing (Perelli-Harris and Styrc, 2018). However, marriage can also be associated with other life transitions as becoming a parent and consequently might present a greater effect on health behaviors (Perelli-Harris and Styrc, 2018).

Little is known about the effect of cohabitation and marriage on lifestyle behaviours and body mass index (BMI) during emerging adulthood; further understanding of these transitions may inform health promotion strategies. We therefore aimed to systematically review the effect of cohabitation and/or marriage on physical activity, diet and BMI changes in the transition between adolescence and emerging adulthood.

## 2 Methods

This review was part of a suite of reviews examining the impact of a number of life transitions (e.g., leaving school, starting work, entering further education, marriage and cohabitation, parenthood) on weight-related outcomes, diet and physical activity over young adulthood. Details of the protocol for this systematic review were registered on PROSPERO (ref: CRD42018106943) and can be accessed at https://www.crd.york.ac.uk/PROSPERO/display_record.php?RecordID=106943.

We conducted a systematic literature search of longitudinal observational studies including data on body mass index, diet and/or physical activity among both sexes from 15 to 35 years of age. Searches were conducted in six databases (MEDLINE, Embase, PsycINFO, Scopus, ASSIA and Web of Science) until July 2019. The full search strategy was focused on three themes: outcomes (weight-related outcomes, physical activity and diet), life transitions (e.g., cohabitation, marriage, parenthood, entering work) and study design (prospective) (Supplementary Table A). The present study focused only on the effect of cohabitation and marriage on physical activity, diet and weight changes during adulthood.

### 2.1 Inclusion criteria

Inclusion was restricted to published longitudinal data with at least two data collection points within the specified age range (i.e., 15–35 years), including marriage and cohabitation, at least one comparison group consistently non-cohabiting and/or non-married, (referred to as single) peers and measurement of a relevant outcome (weight-related outcomes, physical activity or diet). Specifically, cohabitation was defined as couples living together, with intimate relationships, but without legal contractual recognition by the law, while marriage was defined as intimate relationships with legal contractual recognition by the law (Sassler and Lichter, 2020). We sought to include all types of relationship in our search, but most studies did not clarify if the analyses also include same-sex relationships. We set our inclusion criteria to cover the range from age 15 to 35 years to capture transitions occurring from mid-adolescence to emerging adulthood (Levinson, 1986). We set a wide age range for our definition of this transitionary period in order to capture as many studies as possible relevant to our research question, including those from cultures where marriage and cohabitation may occur at young ages. We assumed that participants from studies where baseline measurements occurred during adolescence (e.g. 15 years) were not cohabitating at baseline. All articles published in the English language in a scientific journal regardless of country of origin were considered for inclusion.

To calibrate in/exclusion assessment across reviewers, two groups of three reviewers each independently review a set of 1500 results from the initial title and abstract search (in three sets of 500 per group). Iterative comparison and discussion allowed for development of a consistent screening approach; screening of all remaining titles/abstracts was divided between 5 reviewers. Subsequently, two reviewers independently screened full texts for inclusion and discussed discrepancies to reach consensus. Hand searching of included papers identified three additional full texts, none of which were included after full text screening.

### 2.2 Data extraction

Data extraction was conducted by one author and 100% checked for accuracy by a second reviewer; discrepancies were resolved by discussion. Data extracted included: baseline date (date of the first wave meeting the inclusion criteria), study name, country, ethnicity, sex, socio-economic status; and number of participants, age, outcome and measurement method. Any analysis of difference in change between individuals starting to cohabit or getting married and those consistently single were extracted. Data was extracted for males and females separately where possible. We did not approach authors for additional information as we did not want to bias this review in the event of differential author response (Chan and Altman, 2005).

### 2.3 Assessment of risk of bias

Risk of bias was scored using the Effective Public Health Practice Project Quality Assessment Tool (Armijo-Olivo et al., 2012), which assesses participant representativeness, study size, inclusion and retention rates, quality of outcome data, and quality of change analyses. These criteria were rated by two independent reviewers; in case of disagreement, consensus was derived by discussion with a further reviewer. Each item was scored as ‘strong’, ‘moderate’ or ‘weak’ using pre-specified criteria; if a paper provided insufficient information then it was scored as ‘weak’. Scores for each item were summed and quality was defined as ‘strong’ when up to one item was classified as weak and no more than one item was classified as moderate. Papers were classified as “weak” with two or more “weak” items; other papers were ranked as “moderate”. Scoring details are summarised in Supplementary Table B.

### 2.4 Narrative synthesis

We compared data across the articles according to the outcomes reported (weight-related outcomes, physical activity and diet). Due to a small number of studies included and heterogeneity in the analysis type and coefficients reported, it was not possible to conduct meta-analyses.

## 3 Results

**Fig. 1:**
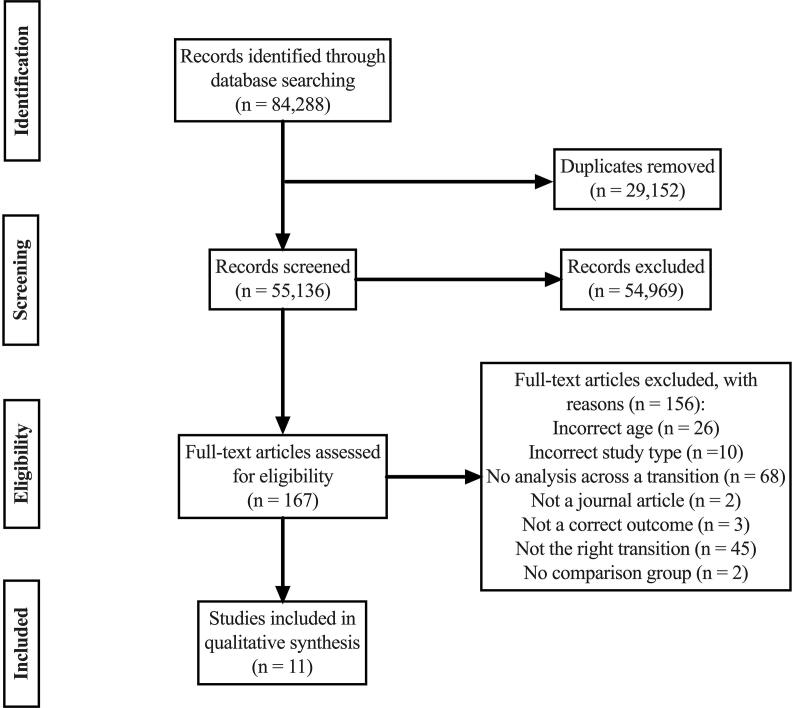
PRISMA flow diagram.

There were 84,288 articles identified in the initial search (including other life events) and 29,152 duplicates were excluded. Titles and abstracts of the remaining 55,136 articles were assessed for inclusion, of which 54,969 papers were excluded. Of the remaining 167 papers taken forward to full text screening, 156 were excluded from the current review for the reasons shown in Fig. 1. This left 11 full text articles exploring the association between cohabitation or marriage and specified health outcomes of interest.

### 3.1 Study characteristics

A summary of all included articles is presented in Table 1. From the 11 articles, outcomes included: weight-related outcomes only (n = 3) (Averett et al., 2008, Bell and Lee, 2005, Chung et al., 2014, Kroeger and Frank, 2018), physical activity only (n = 4) (Bell and Lee, 2005, Brown et al., 2009, Hull et al., 2010, Miller et al., 2019), diet only (n = 2) (Smith et al., 2017, Winpenny et al., 2018), weight-related outcomes and physical activity (n = 1) (The and Gordon-Larsen, 2009), and all outcomes (n = 1) (Burke, 2004). Two articles only included female participants (Bell and Lee, 2005, Brown et al., 2009); all others included both men and women. The sample size ranged between 405 and 11,766 participants. Follow-up length ranged between 2 and 17 years. Four studies included separate analyses for cohabitation and marriage (Averett et al., 2008, Bell and Lee, 2005, Kroeger and Frank, 2018, The and Gordon-Larsen, 2009). All studies were classified at having at least one risk of bias criteria classified as “weak”. Of the 11 studies included, nine were classified as “weak” regarding recruitment indicating difficulties with recruitment and retention of this population. Also, there was one study that assessed the BMI trajectory considering the years of marriage and found that the increase trend occurred especially one year after getting married (Averett et al., 2008).

**Table 1:** Summary of articles analysing the effect of becoming married/cohabitating on health outcomes (not married/cohabitating during baseline).

References by outcome	Year	Country	Participants (N)	Sex	SES	Follow-up (years)	Quality assessment	Measures	Main findings
*BMI*									
Averett et al.	2008	USA	10,423	Both	Number of education years: women: 12.9y ± 2.3. Men: 12.7y ± 2.4.	17	Weak	BMI (self-reported stature and body mass) – treated continuously	Getting married/cohabitating was associated with a lower BMI among women, but not men, when compared to a ‘consistently single’ group (LOG) in adjusted models: **Men:** starting cohabitating: *B* = −0.007, *p* < 0.05; getting married: *B* = 0.029, *p* < 0.01 **Women:** starting cohabitating: *B* = −0.018, *p* < 0.01; getting married: *B* = − 0.025, *p* < 0.01
Burke*	2004	Australia	405	Both	N/A	7	Weak	BMI (measured stature and body mass) – treated continuously	Living with a partner was associated with an increase in BMI for both sexes compared to a ‘consistently single’ group (*p* = 0.042). **Men:** mean change in BMI –kg/m^2^ (SE): family home: 2.2(0.3), away of home, without partner: 2.4(0.3), with partner: 2.8(0.4) **Women:** mean change in BMI –kg/m^2^ (SE): family home: 1.5(0.4), away of home, without partner: 1.1(0.5), with partner: 2.1(0.3)
Chung et al.	2014	USA	9222	Both	No college degree (%): Healthy/healthy: Men:67.4%, women: 52.6%, Healthy–Overweight/Obese: Men:69.3%, women: 68.1%. Overweight/Obese- Overweight/Obese: Men:78.4%, women: 75.3%, Overweight/Obese–Healthy: Men: 82.9%, women: 66.6%	12	Strong	BMI (measured stature and body mass) – treated as categorial (healthy, overweight or obese)	Having been married was associated with a higher likelihood of becoming overweight compared to a ‘consistently single’ group **Whole sample:** OR = 1.4, 95% CI: 1.2, 1.6; **Men:** OR = 1.6, 95% CI: 1.3, 2.0; **Women:** OR = 1.2, 95% CI: 1.0, 1.5.
Kroeger & Frank	2018	USA	11,766	Both	At least college degree: White: Men: 32%, women: 41%. Black: Men: 19%, women: 28%. Hispanic: Men: 19%, women: 26%. Asian: Men: 55%, women: 52%. Multiracial: Men: 31%, women: 33%.	13	Strong	BMI (measured stature and body mass) – treated continuously	Getting married, but not starting cohabitating, was associated with higher BMI when adopting ‘consistently single’ as the reference group: **Men:** starting cohabitating: *B* = 0.31, p < 0.10; getting married: *B* = 0.47, *p* < 0.001 **Women:** starting cohabitating: *B* = 0.03, *p* > 0.10; getting married: *B* = 0.55, *p* < 0.001.
The & Gordon-Larsen	2009	USA	6949	Both	<12 years of education (%): Single/dating-dating: 6.8%. Single/dating-single: 9.7%. Single/dating-cohabitating: 18.2%. Single/dating-married: 16.8%.	5–6	Weak	BMI (self-reported stature and body mass) – treated as categorial (non-obese vs. obese)	Marriage was associated with incidence of obesity compared to those dating. Cohabitating was also associated with incidence of obesity in women, but not men. **Men:** starting cohabitating: OR = 1.30, 95% CI (0.81, 2.09); getting married: OR = 2.07, 95% CI (1.33, 3.25); **Women:** starting cohabitating: OR = 1.63, 95% CI (1.14, 2.32); getting married: OR = 2.27, 95% CI (1.55, 3.34).

*PA*									
Bell et al.	2005	Australia	8545	Women	N/A**	4	Weak	Self-reported PA with specific questionnaire – treated as categorical (inactive vs. active)	Getting married and starting cohabitation was associated with decreased PA compared to remaining single: **Women:** starting cohabitating: OR = 1.4, 95% CI (1.2, 1.7); getting married: OR = 1.6, 99% CI (1.3, 2.0).
Brown et al.	2009	Australia	7173	Women	Highest level of education: No formal education: 1.4%. Year 10–12: 32.6%. Trade, certificate, college, university: 24.6%	3	Weak	Self-reported PA with specific questionnaire – treated as categorial (maintained, decrease or increase).	Getting married/cohabitating was associated with higher odds of decreasing PA and was not associated with increasing PA compared to those who remained single. **Women:** decreasing PA: OR = 1.32, 95% CI (1.14, 1.52); increasing PA: OR = 0.95, 95% CI (0.79, 1.14).
Burke*	2004	Australia	405	Both	N/A	7	Weak	7-day recall – treated as categorial (inactive vs. active)	Men with a partner had higher rates of physical inactivity (*p* = 0.003 for sex * partner * inactivity) when compared with those who remained single. **Men:** prevalence of physical inactivity: family home: 18y: 23%, 25y: 41%; away of home, without partner: 18y: 13%, 25y: 34%; with partner: 18y: 24%, 25y: 53%. **Women:** prevalence of physical inactivity: family home: 18y: 47%, 25y: 36%; away of home, without partner: 18y: 42%, 25y: 43%; with partner: 18y: 44%, 25y: 45%.
Hull et al.	2010	USA	638	Both	52% had at least a college degree	2	Moderate	Past Year Leisure Time Physical Activity Questionnaire – treated continuously (change in h/week)	PA differences were not different among participants that stayed single and participants that became married or started cohabitating: **Whole sample**: mean change (SD): stayed single: −1.2 (7.6) vs. became married/cohabitating: −0.7 (6.2); *p* = 0.70; **Men:** mean change (SD): stayed single: −1.9 (7.6) vs. became married/cohabitating: −2.3 (8.0); *p* = 0.62. **Women:** mean change (SD): stayed single: −0.2 (7.7) vs. became married/cohabitating: 0.1 (4.2); *p* = 0.65.
Miller et al.	2019	USA	2287 for 1st period and 1830 for 2nd period	Both	Socioeconomic status: Low: 11.8%. Low-middle: 16.0%. Middle: 25.8%. High-middle: 28.7%. High: 17.7%.	5–6 years for 1st period and 7–8 years 2nd period	Weak	Self-reported PA with specific questionnaire – treated continuously (change in h/week)	Cohabitation/marriage was associated with a reduced physical activity only among women during the second period of follow-up when compared with participants that remained single: **Men:** 1st period: *B* = 0.19, 95% CI: −0.83, 1.21. 2nd period: *B* = −0.64, 95%CI: −1.55, 0.27. **Women:** 1st period: *B* = −0.55, 95%CI:-1.23, 0.13. 2nd period: *B* = −1.60, 95%CI: −2.23, −0.98.
The & Gordon-Larsen	2009	USA	6949	Both	<12 years of education (%): Single/dating-dating: 6.8%. Single/dating-single: 9.7%. Single/dating-cohabitating: 18.2%. Single/dating-married: 16.8%.	5–6	Weak	Self-reported PA with specific questionnaire – treated as categorial (≥2 bouts/week vs. ≤2 bouts/week)	Prevalence of MVPA was lower among married participants when compared to dating among men but not women. **Men:** prevalence of MVPA (SE): getting married: 36.6 (0.02); starting cohabitating: 40.3 (0.02); dating: 45.9 (0.02). p ≤ 0.05 for married vs. dating. **Women:** prevalence of MVPA (SE): getting married: 25.0 (0.02); starting cohabitating: 30.3 (0.02); dating: 35.0 (0.02). p > 0.05 for married vs. dating.

*Diet*									
Smith et al.	2017	Australia	1402	Both	50.2% college degree, 23.5% vocational, 26.4% high school or less.	5	Moderate	127-item Food Frequency Questionnaire and a Food Habits Questionnaire – treated continuously (dietary guideline index scores)	Starting cohabitating/get married was not associated with diet quality in both men and women when compared the mean difference against peers that remained single. **Men:** mean difference: −2.07, 95% CI: −6.73, 2.58 **Women:** mean difference: −2.81, 95% CI: −6.44, 0.82
Burke*	2004	Australia	405	Both	N/A	7	Weak	3-day diet record – treated continuously (mean difference in the energy intake – MJ)	Significant sex-by-partner interaction in predicting mean differences of total energy intake (*p* = 0.024) when comparing with participants that remained single. The increase was higher among women compared to men: **Men:** mean difference: With partner = −1.2 MJ, away of home, without partner = −2.7 MJ, family home = −0.7 MJ **Women:** mean difference: With partner = 1.0 MJ, away of home, without partner = 0.1 MJ, family home = −0.2 MJ
Winpenny et al.	2018	Norway	1100	Both	Parental education during the baseline: 40.3% college/university, 44.7% secondary school and 14.9% primary school only	1–15	Weak	Specific questionnaire with frequency of consumption of fruit, vegetables, sweets/chocolate and sugar-containing soft drinks ingestion – treated continuously (frequency of intake)	Starting cohabitation/marriage was not associated with changes in the weekly frequency consumption of fruit, vegetables, sweets/chocolate and sugar-containing soft drinks when comparing against those that remained single. **Men:** fruit: *B* = 0.50, 95% CI: −0.27, 1.27; vegetables: *B* = 0.24, 95% CI: −0.41, 0.89; confectionery: *B* = 0.01, 95% CI: −0.52, 0.55; sugar-sweetened beverages: *B* = − 0.34, 95% CI: −1.06, 0.38. **Women:** fruit: *B* = 0.42, 95% CI: −0.17, 1.02; vegetables: *B* = 0.28, 95%CI: −0.59, 0.14; confectionery: *B* = −0.22 95% CI: −0.59, 0.14; sugar-sweetened beverages: *B* = 0.20 95% CI: −0.25, 0.65.

Abbreviations: SES, socioeconomic status; BMI, body mass index; MVPA, moderate-to-vigorous physical activity; PA, physical activity; N/A, not available. *Burke, 2004 divided participants into three groups: 1) participants starting cohabitation/getting married, 2) participants living in the family home (family home), and 3) those living outside the family home (away no partner).”

Considering the whole sample of each study, all studies including BMI as an outcome found increases in BMI (Averett et al., 2008, Burke, 2004, Chung et al., 2014, Kroeger and Frank, 2018, The and Gordon-Larsen, 2009). For example, Burke (2004) found a mean increase of 2.5 ± 2.0 kg/m^2^ for men and 1.7 ± 2.9 kg/m^2^ among women, between 18 and 25 years. On the other hand, the majority of the studies found that physical activity levels were stable for the whole group overtime (Bell and Lee, 2005, Brown et al., 2009, Hull et al., 2010), with only two studies pointing a slight trend for decreasing overtime for the whole sample (Miller et al., 2019, The and Gordon-Larsen, 2009) and one study showing a decrease only among men (Burke, 2004). For diet, the findings were mixed with Smith et al. (2017) showing an increase in the diet quality, while Burke (2004) found that the total energy intake decreased among men and increased among women. Also, Winpenny et al. (2018) found a U-shape trajectory for the consumption of fruits and vegetables, with a decreasing trend between 15 and 20 years and an increasing trend between 20 and 30 years.

### 3.2 BMI

Of five articles that included BMI as the outcome, three studies included measured body mass and stature (Burke, 2004, Chung et al., 2014, Kroeger and Frank, 2018), while two studies included self-reported measures (Averett et al., 2008, The and Gordon-Larsen, 2009). All included a follow-up of at least five years and three studies had a follow-up longer than 10 years (Averett et al., 2008, Chung et al., 2014, Kroeger and Frank, 2018). Three studies examined marriage and cohabitation separately (Averett et al., 2008, Kroeger and Frank, 2018, The and Gordon-Larsen, 2009). Averett et al. (2008) found that women getting married as well as those starting cohabitation and men starting cohabitation presented lower BMI than consistently single participants, while men getting married presented higher BMI than consistently single participants. The other two studies found that getting married, but not cohabitation, was associated with increased BMI, compared to remaining single (Kroeger and Frank, 2018, The and Gordon-Larsen, 2009). The two studies analysing cohabitation and marriage as a single indicator demonstrated an association with higher BMI when compared with the consistently single group (Burke, 2004, Chung et al., 2014). For example, compared to remaining single, Chung et al. (2014) reported increased long term risk for overweight after getting married, (OR for men = 1.6, 95% CI: 1.3 to 2.0; OR for women = 1.2, 95% CI: 1.0 to 1.5) with The et al. (2009) reporting increased risk for obesity for men and women after marriage (OR for men = 2.07, 95% CI: 1.33 to 3.25; OR for women = 2.27, 95% CI: 1.55 to 3.34).

### 3.3 Physical activity

All six studies reporting on physical activity included a self-reported measure (Bell and Lee, 2005, Brown et al., 2009, Burke, 2004, Hull et al., 2010, Miller et al., 2019, The and Gordon-Larsen, 2009). Three studies presented a follow-up longer than five years (Burke, 2004, Miller et al., 2019, The and Gordon-Larsen, 2009) but none followed-up for more than 10 years.

Four of the six studies among women reported that cohabitation or marriage was associated with greater decreases in physical activity compared to remaining single (Bell and Lee, 2005, Brown et al., 2009, Burke, 2004, Miller et al., 2019). Two of four studies among men found that starting cohabitation or getting marriage was associated with a larger decrease in physical activity compared to remaining single (Burke, 2004, The and Gordon-Larsen, 2009) (Table 2**)**. Two of the studies included compared the effect of getting married and starting cohabitation on physical activity (Bell and Lee, 2005, The and Gordon-Larsen, 2009), and found no difference between the two transitions with both associated with greater declines in physical activity.

**Table 2:** Summary of study findings.

Outcome	Sex	Association		
		Significant decrease	Null	Significant increase
BMI				
(5 studies)	***Men***			
	Cohabitation	Averett et al., 2008	Kroeger and Frank, 2018, The and Gordon-Larsen, 2009	
	Marriage	None		Averett et al., 2008, Chung et al., 2014, Kroeger and Frank, 2018, The and Gordon-Larsen, 2009
	Cohabitation or marriage	None		Burke, 2004
	***Women***			
	Cohabitation	Averett et al., 2008	Kroeger & Frank, 2018	The & Gordon-Larsen, 2009
	Marriage	Averett et al., 2008		Chung et al., 2014, Kroeger and Frank, 2018, The and Gordon-Larsen, 2009
	Cohabitation or marriage	None		Burke, 2004

PA				
(6 studies)	***Men***			
	Cohabitation		The & Gordon-Larsen, 2009	None
	Marriage	The & Gordon-Larsen, 2009		None
	Cohabitation or marriage	Burke, 2004	Hull et al., 2010, Miller et al., 2019	None
	***Women***			
	Cohabitation	Bell & Lee, 2005	The & Gordon-Larsen, 2009	None
	Marriage	Bell & Lee, 2005	The & Gordon-Larsen, 2009	None
	Cohabitation or marriage	Brown et al., 2009, Miller et al., 2019	Burke, 2004, Hull et al., 2010	None

Diet				
(3 studies)	***Men***			
	Cohabitation	None	None	None
	Marriage	None	None	None
	Cohabitation or marriage	None	Burke, 2004, Smith et al., 2017	None
	***Women***			
	Cohabitation	None	None	None
	Marriage	None	None	None
	Cohabitation or marriage	None	Smith et al., 2017, Winpenny et al., 2018	Burke, 2004 (energy intake)

**Note.** BMI, body mass index; PA, physical activity.

### 3.4 Diet

From the three studies that included diet as an outcome, two included a follow-up period longer than five years and reported data on men and women (Burke, 2004, Smith et al., 2017). Moreover, one study reported data on multiple time points (Winpenny et al., 2018). Burke et al. (2004) found that starting cohabitation and/or marriage was associated with an increase in total energy intake, especially among women, when comparing to the consistently single group. However, Smith et al., 2017, Winpenny et al., 2018 found no association between starting cohabitation and diet quality among both sexes.

## 4 Discussion

### 4.1 Main findings

Emerging adulthood is a critical period marked by several life events that can cause changes in health behaviours (Corder et al., 2017, Harris et al., 2009, Wethington, 2005, Winpenny et al., 2017). This systematic review aimed to investigate whether starting cohabitation and/or marriage is associated with changes in BMI, physical activity and diet, and identified 11 studies. There was consistent evidence that getting married was associated with greater increases in BMI among both men and women, but the evidence for cohabitation and change in BMI was more mixed. Similarly, the association of marriage and cohabitation with physical activity was mixed, with approximately half of the articles finding an association with decreasing physical activity and the other half finding null associations. The limited information available regarding diet did not provide evidence of a change in diet after starting cohabitation or marriage.

### 4.2 Relationship to prior knowledge

Cohabitation and marriage are likely to lead to changes in the social environment, particularly in the home, but also potentially more widely, such as changing groups of friends. It is likely that some of these social changes may lead to increases in energy intake and reductions in energy expenditure which could be linked to changes in BMI (Perry et al., 2016). The increased social responsibilities of cohabitation and especially marriage have been associated with the adoption of unhealthy behaviours, for example, lower physical activity could be due to reduced leisure time (Nomaguchi and Bianchi, 2004). Cohabitation and marriage could also influence diet through increased consumption of regular meals (Marshall and Anderson, 2002), in larger portions (Worsley, 1988), which has been associated with higher weight status (Mata et al., 2018).

However, we did not find a consistent association between cohabitation or marriage and diet. There are some issues that can partly explain this incoherence, as the low number of studies, which analysed different indicators of diet, such as an index of diet quality based on dietary intake collected using a food frequency questionnaire (Smith et al., 2017), total energy intake estimated through a 3-day diet record (Burke, 2004) and the intake of specific groups of food (Winpenny et al., 2018). Moreover, there could be other pathways for increasing BMI after cohabitation; a higher partner acceptance of weight status after marriage has been proposed as a potential psychological pathway to explain weight gain after marriage (Bove and Sobal, 2011, Perry et al., 2016). Therefore, future research should investigate potential mediators for the association between cohabitation and BMI, including physical activity, diet and psychological factors.

Cohabitation and marriage have previously been suggested to have different impacts on diet for men and women and for various dietary components (Conklin et al., 2014, Perry et al., 2016, Vinther et al., 2016). Previous research has shown that marriage is associated healthy dietary behaviours in older men, such as increased fruit intake (Conklin et al., 2014, Vinther et al., 2016). Conversely, cohabitation could also be associated with poor behaviours, particularly among women, such as elevated energy intake due to cultural conventions including the preparation of more elaborate meals on a regular basis (Perry et al., 2016).

### 4.3 Cohabitation or Marriage?

Four articles analysed marriage and cohabitation separately (Averett et al., 2008, Bell and Lee, 2005, Kroeger and Frank, 2018, The and Gordon-Larsen, 2009). Together, these show consistent evidence of greater increases in BMI among people getting married (with exception for women in the Averett et al, 2008 study), while cohabitation was not consistently associated with changes in BMI, compared with remaining single (Averett et al., 2008, Kroeger and Frank, 2018, The and Gordon-Larsen, 2009), especially among men. However, both studies that separated marriage and cohabitation when examining the impact on physical activity, found no differences between cohabitation and marriage (Bell and Lee, 2005, The and Gordon-Larsen, 2009). Although cohabitation and marriage could both be characterized as beginning to live with a partner, there are some key differences. As the majority of weddings are preceded by periods of cohabitation (Beaujouan & Ní Bhrolcháin, 2011), the latency period of living with a partner among cohabitating couples is probably shorter than among married couples and consequently, the effect of living with a partner can be time-dependent (Mata et al., 2018). Marriage can promote greater social and legal stability than cohabitation, which may also improve mental wellbeing (Perelli-Harris and Styrc, 2018), which may reflect a greater acceptability of increasing adiposity. Compared to cohabitation, marriage is associated with higher rates of childbirth; it has been proposed that becoming a parent could explain some of the negative effect of marriage on health (Perelli-Harris and Styrc, 2018).

### 4.4 Different associations of cohabitation with BMI, physical activity and diet

On average BMI is known to increase over the transition from adolescence into adulthood, while physical activity is reported to decrease and diet shows multiple changes in different components independent of cohabitation status (Caman et al., 2013, Christoph et al., 2019, Corder et al., 2017, Winpenny et al., 2017). However, as previously discussed, this review suggested that getting married/starting cohabitation had a more consistent influence on BMI, than diet and physical activity. There are many possible explanations for this. Firstly, BMI is relatively reliable to measure, easier to assess using consistent methods and is more regularly used in different cohorts. Conversely, diet and physical activity, are often assessed using subjective questionnaires, are multi-faceted behaviours, are more likely to change overtime and consequently are less often used in classical cohort studies (Bingham et al., 1994, Shephard, 2003). Moreover, as physical activity and diet are behaviours, they are more likely to have higher variability with time as well as a wider range of determinants in comparison to a biological factor like BMI (Bauman et al., 2012, Hill and Melanson, 1999, Popkin, 2011). It is also possible that getting married can increase the acceptability of higher adiposity, with a greater latency period than cohabitation, and should be another stronger determinant of a higher BMI, but not necessarily for risk behaviors as physical activity and diet, which can be promoted by the higher social support and control of health behaviors when married (Schoeppe et al., 2018, Umberson, 1992).

Although the consistency of findings from studies of physical activity was less than that seen for BMI, several studies showed that starting cohabitation/getting married was also associated with reduced physical activity, while no study found the opposite, suggesting that reductions in physical activity may be an important pathway to increases in BMI over this transition. This is consistent with evidence regarding the role of physical activity in energy balance and prevention of weight gain from adolescence to adulthood (Jakicic et al., 2019, Parsons et al., 2006).

### 4.5 Strengths and limitations

We identified weaknesses in the underlying evidence base. Only two of the included studies were classified as being high quality. The most consistent risk of bias was found among the items assessing recruitment, retention and methodology (e.g., use of self-report methods), which are common problems of cohort studies (Teague et al., 2018). Few included studies included follow-up periods longer than 5 years, and none had long term follow up for studies assessing physical activity or diet. Therefore, studies with long-term follow up investigating the effect of cohabitation on physical activity and diet are still warranted.

To our knowledge, this is the first systematic review investigating the effect of cohabitation on BMI, physical activity and diet in the transition between adolescence and emerging adulthood, compared to adults remaining single. We applied accepted systematic review methods, including searching six databases, double full-text screening and quality assessment and 100% checking of data extraction.

The present review also has some limitations. Firstly, due to the heterogeneity of methods and type of analysis, it was not possible to conduct meta-analysis. However, we concisely summarised previous findings and inferred potential trends. Secondly, we assumed that participants with a baseline measurement during adolescence were not cohabitating, but it is possible that some participants were already cohabitating at baseline. Also, some studies used self-reported stature and body mass for BMI estimation, which tends to lead to an underestimation of BMI (McAdams et al., 2007). It is not known whether this bias is differential across the exposure groups of interest. Third, the majority of the studies did not state if they included same-sex couples, which can be a limitation, especially considering that some studies were conducted before the legalization of same-sex marriage in different countries. Fourth, considering the age-range, it is plausible that the participants were also experiencing concomitant life transitions that can also affect BMI, physical activity and diet (e.g. parenthood, education and employment transitions) and these were not controlled for in the majority of the studies (Corder et al., 2020, Winpenny et al., 2020). Fifth, BMI, physical activity and diet change with age and the effect of cohabitation on these indicators can vary with age even inside the transition between adolescence and early adulthood. Sixth, most studies did not include the age at which participants started cohabitating or were married. This can introduce bias, considering that some studies adopted a relatively long follow-up period and consequently, it is possible to have participants with different marriage or cohabitation durations grouped together.

### 4.6 Implications for policy, practice and research

The current systematic review suggests that individuals who get married during emerging adulthood are at risk for increasing BMI and could be targeted for obesity prevention during emerging adulthood. In this sense, interventions aiming to reduce BMI should be focused among people who get marriage during the period between adolescence and emerging adulthood. The current study highlights some areas that warrant further investigation in future studies. First, there is an urgent need for studies of higher methodological quality as only two of the included studies were of high quality of evidence. Second, more studies analysing the association between cohabitation and BMI are warranted as this association was not consistent. Third, there is still limited evidence of the association of cohabitation and marriage with physical activity and diet, especially in studies including men. Fourth, none of the studies measuring physical activity used device-based measures (e.g., accelerometry), which should be considered in future investigations. Finally, studies with longer follow-up periods and more frequent data collection would be helpful to better understand the impact of cohabitation on physical activity and diet.

## 5 Conclusion

The evidence from the present systematic review consistently suggests that getting married was associated with greater increases in BMI during emerging adulthood among men and women than remaining single, although the evidence for cohabitation was mixed. The start of marriage may be an important opportunity for weight management interventions. There was limited evidence for the effect of cohabitation and marriage on physical activity and diet, and this requires further research. To progress existing knowledge, future research should be of high methodological quality with frequent data collection using validated measures of the behaviour.

## Funding

André O Werneck is supported by the 10.13039/501100001807São Paulo Research Foundation (FAPESP) (FAPESP process: 2018/19183-1). Funding for this study and the work of all authors was supported, wholly or in part, by the Centre for Diet and Activity Research (CEDAR), a UKCRC Public Health Research Centre of Excellence (RES-590-28-0002). Funding from the British Heart Foundation, Department of Health, Economic and Social Research Council, Medical Research Council, and the Wellcome Trust, under the auspices of the UK Clinical Research Collaboration, is gratefully acknowledged. The work of Kirsten Corder, Eleanor Winpenny and Esther M F van Sluijs was supported by the Medical Research Council (MC_UU_12015/7).

## Declaration of Competing Interest

The authors declare that they have no known competing financial interests or personal relationships that could have appeared to influence the work reported in this paper.
