# Supplementary material for: Cohabitation and marriage during the transition between adolescence and emerging adulthood: A systematic review of changes in weight-related outcomes, diet and physical activity
Source: Prev Med Rep. 2020 Nov 28;20:101261. doi: 10.1016/j.pmedr.2020.101261 (PMC7736988; doi:10.1016/j.pmedr.2020.101261)
Supplement: Supplementary data 1 [file mmc1.doc]

**Supplementary Table A.** Search strategy.

| 1 | Diet Outcomes  Physical activity outcomes  Health outcomes | food*[Title/Abstract] OR beverage*[Title/Abstract] OR nutrient*[Title/Abstract] OR macronutrient*[Title/Abstract] OR “energy intake”[Title/Abstract] OR diet[Title/Abstract] OR diets[Title/Abstract] OR “dietary”[Title/Abstract] OR nutrition[Title/Abstract] OR nutritional[Title/Abstract] OR fruit[Title/Abstract] OR vegetable[Title/Abstract] OR fruits[Title/Abstract] OR vegetables[Title/Abstract] OR dairy[Title/Abstract] OR grain*[Title/Abstract] OR meat[Title/Abstract] OR cereal*[Title/Abstract] OR “soft drink*”[Title/Abstract] OR soda[Title/Abstract] OR SSB[Title/Abstract] OR SSBs[Title/Abstract] OR salt[Title/Abstract] OR sugar*[Title/Abstract]  OR "Food"[Mesh] OR "Beverages"[Mesh] OR diet[Mesh] OR "Nutrition Surveys"[Mesh] OR "Diet Records"[Mesh] OR "Dietary Fats"[Mesh] OR "Dietary Proteins"[Mesh] OR "Dietary Carbohydrates "[Mesh] OR "Healthy Diet"[Mesh]  OR cook*[Title/Abstract] OR “food preparation”[Title/Abstract] OR “eating behaviour”[Title/Abstract] OR “eating behavior”[Title/Abstract] OR meal*[Title/Abstract] OR snack*[Title/Abstract] OR “dining out”[Title/Abstract] OR breakfast[Title/Abstract] OR dinner[Title/Abstract] OR lunch[Title/Abstract] OR supper[Title/Abstract] OR “fast food”[Title/Abstract] OR “fast-food”[Title/Abstract] OR restaurant[Title/Abstract] OR “take-away”[Title/Abstract] OR takeaway[Title/Abstract] OR “eating out”[Title/Abstract] OR “away from home”[Title/Abstract] OR “out of home”[Title/Abstract] OR “away-from-home”[Title/Abstract] OR “out-of-home”[Title/Abstract] OR “home-cook*”[Title/Abstract] OR “home prepar*”[Title/Abstract] OR “home cook*”[Title/Abstract] OR “home-prepar*”[Title/Abstract]  OR "physical activity"[Title/Abstract] OR "physical activities"[Title/Abstract] OR “physically active”[Title/Abstract] OR "active transport"[Title/Abstract] OR "active travel"[Title/Abstract] OR exercise*[Title/Abstract] OR cycle[Title/Abstract] OR cycling[Title/Abstract] OR walk*[Title/Abstract] OR sport*[Title/Abstract] OR "energy expenditure"[Title/Abstract] OR MVPA[Title/Abstract] OR “vigorous activity”[Title/Abstract] OR VPA[Title/Abstract] OR “moderate activity” [Title/Abstract] OR “light activity”[Title/Abstract] OR MPA[Title/Abstract] OR “intensity activity”[Title/Abstract] OR “LPA”[Title/Abstract] OR “strenuous activity”[Title/Abstract] OR “Exercise”[MeSH] OR “Sports”[Mesh]  OR “BMI”[Title/Abstract] OR "weight gain"[Title/Abstract] OR "weight loss"[Title/Abstract] OR "body weight"[Title/Abstract] OR "body mass"[Title/Abstract] OR "change in weight"[Title/Abstract] OR " weight change"[Title/Abstract] OR "waist circumference"[Title/Abstract] OR anthropometry[Title/Abstract] OR anthropometric[Title/Abstract] OR “fat mass”[Title/Abstract] OR “lean mass”[Title/Abstract] OR “body composition”[Title/Abstract] OR skinfold[Title/Abstract] OR overweight[Title/Abstract] OR obesity[Title/Abstract] OR adiposity[Title/Abstract] |
| --- | --- | --- |
| 2 | Longitudinal | longitudinal[Title/Abstract] OR cohort[Title/Abstract] OR prospective[Title/Abstract] OR “follow-up*”[Title/Abstract] OR "follow up"[Title/Abstract] OR "Follow-Up Studies"[Mesh] OR “Prospective Studies”[Mesh] OR “Longitudinal Studies”[Mesh] OR “Cohort Studies”[Mesh] OR “life-course”[Title/Abstract] OR “life course”[Title/Abstract] OR “repeated measure”[Title/Abstract] OR “repeated-measure”[Title/Abstract] |
| 3 | Transition | “Further education”[Title/Abstract] OR “Higher education”[Title/Abstract] OR “entering education”[Title/Abstract] OR “leaving education”[Title/Abstract] OR “leaving school”[Title/Abstract] OR “school leaver*”[Title/Abstract] OR college[Title/Abstract] OR university[Title/Abstract] OR freshman[Title/Abstract] OR freshmen[Title/Abstract] OR Graduate[Title/Abstract] OR Undergrad*[Title/Abstract] OR employment[Title/Abstract] OR “first job”[Title/Abstract] OR “starting work” [Title/Abstract] OR “moving house”[Title/Abstract] OR “change of address”[Title/Abstract] OR “residential relocation”[Title/Abstract] OR “residential mobility”[Title/Abstract] OR “residential instability”[Title/Abstract] OR “geographic* mobility”[Title/Abstract] OR “geographic relocation”[Title/Abstract] OR “residential relocation”[Title/Abstract] OR “residential stability”[Title/Abstract] OR  OR “living arrangements”[Title/Abstract] OR cohabit*[Title/Abstract] OR marriage[Title/Abstract] OR “marital status” [Title/Abstract] OR parity[Title/Abstract] OR parous[Title/Abstract] OR childbearing[Title/Abstract] OR postpartum[Title/Abstract] OR “post-partum”[Title/Abstract] OR "first child*"[Title/Abstract] OR "having children"[Title/Abstract] OR "having a child"[Title/Abstract] OR trajector*[Title/Abstract] OR “transition*”[Title/Abstract] OR “life transition”[Title/Abstract] OR “life events”[Title/Abstract] OR “Life Change Events"[Mesh] OR "Marital Status"[Mesh] OR "Employment"[Mesh] |
| 4 | Additional filters | English[lang]  NOT Review[ptyp]  NOT Neoplasm[Mesh]  Humans (filter) |
| 5 |  | 1 AND 2 AND 3 AND 4 |

**Supplementary Table B**. Risk of bias scoring criteria, adapted from the Effective Public Health Practice Project Quality Assessment Tool.

| **Characteristic** | **Question** | **Scoring** | **Scoring** |
| --- | --- | --- | --- |
| **Representativeness** | 1) Are the individuals selected to participate in the study likely to be representative of the target population? | 1 Very likely  2 Somewhat  3 Not likely  4 Can’t tell | Strong  Moderate  Weak  Weak |
|  | 2) What percentage of selected individuals agreed to participate? | 1 80-100%  2 60–79%  3 <60%  4 Not applicable  5 Can’t tell | Strong  Moderate  Weak  Weak  Weak |
| **Number of participants** | 3) How many participants were in the study? | 1 >1000  2 999-101  3 <100 | Strong  Moderate  Weak |
| **Drop-outs** | 4) Were withdrawals and drop-outs reported in terms of numbers and/or reasons per group? | 1 Yes  2 No  3 Can’t tell  4 Not Applicable | Strong  Moderate  Weak  Weak |
|  | 5) Indicate the percentage of participants completing the study. (If the percentage differs by groups, record the lowest). | 1 80-100%  2 60–79%  3 <60%  4 Not applicable  5 Can’t tell | Strong  Moderate  Weak  Weak  Weak |
| **Data collection** | 6) Was the tool objective or subjective? | 1 Objective  2 Reported  3 Can’t tell | Strong  Moderate/Weak  Weak |
|  | 7) Was the tool valid? | 1 Yes  2 No  3 Can’t tell | Strong/Moderate  Weak  Weak |
|  | 8) Was the tool reliable? | 1 Yes  2 No  3 Can’t tell | Strong/Moderate  Weak  Weak |
|  | 9) Was the tool the same at all time-points? | 1 Yes  2 No  3 Can’t tell | Strong-Weak  Weak |
| **Analyses** | 10) Was change in outcome statistically tested? | 1 Yes  2 No  3 Can’t tell | Strong/Moderate  Weak  Weak |
|  | 11) Was adjustment for potential confounders included? | 1 Yes  2 No  3 Can’t tell | Strong/Moderate  Moderate/Weak  Weak |
| **Note.** When multiple questions represent one category, the results of all category questions were combined to obtain a score and the lowest ranking for a category was taken. Each item was scored as ‘strong’, ‘moderate’ or ‘weak’ using pre-specified criteria; if a paper provided insufficient information then it was scored as ‘weak’. Scores for each item were summed and quality was defined as ‘strong’ when up to one item was classified as weak and no more than one item was classified as moderate. Papers were classified as “weak” with two or more “weak” items; other papers were ranked as “moderate”. | | | |
